# Fertility History and Cognition in Later Life

**DOI:** 10.1093/geronb/gbw013

**Published:** 2016-02-29

**Authors:** Sanna L Read, Emily M D Grundy

**Affiliations:** 1 Department of Social Policy, London School of Economics and Political Science, UK.

**Keywords:** Cognitive functioning, Older age, Parity, Timing of births

## Abstract

**Objectives:**

To investigate the association between fertility history and cognition in older men and women.

**Method:**

We analyzed associations between number of children (parity) and timing of births with level and change in cognition among 11,233 men and women aged 50+ in England using latent growth curve models. Models were adjusted for age, socioeconomic position, health, depressive symptoms, control, social contacts, activities, and isolation.

**Results:**

Low (0–1 child) and high parity (3+ children) compared to medium parity (2 children) were associated with poorer cognitive functioning, as was an early age at entry to parenthood (<20 women/23 men). Many of these associations disappeared when socioeconomic position and health were controlled. For women, however, adjusting for socioeconomic position and social contacts strengthened the association between childlessness and poor cognition. Late motherhood (>35) was associated with better cognitive function.

**Conclusion:**

Associations between fertility history and cognition were to large extent accounted for socioeconomic position, partly because this influenced health and social engagement. Poorer cognition in childless people and better cognition among mothers experiencing child birth at higher ages suggest factors related to childbearing/rearing that are beneficial for later cognitive functioning, although further research into possible earlier selection factors is needed.

Aging is associated with declines in cognitive functioning, but there is considerable heterogeneity between individuals in this process, most probably reflecting a range of environmental and behavioral influences over the life course as well as genetic influences (Deary et al., 2009). In this study, we examine whether one domain of major significance in most people’s lives—fertility history—is associated with level and change in cognitive functioning in later life. The timing of parenthood and number of children born have important implications for activities, roles, social and economic resources, and lifestyles throughout adulthood (Knoester & Eggebeen, 2006; Nomaguchi & Milkie, 2003; Wenger, Dykstra, Melkas, & Knipscheer, 2007)—all domains which may directly or indirectly affect cognitive reserve and later life cognitive functioning (Agrigoroaei & Lachman, 2011; Brunner, 2005). Moreover, previous research has identified links between fertility histories and later life physical health (Grundy & Read, 2015), which itself associated with cognitive function (Brunner, 2005). We use nationally representative longitudinal data from England to investigate the association between fertility history and later life cognitive functioning in women and men. Unlike most previous studies on this topic which have focused on the possible role of differential exposures to estrogen and other hormones in women (Behl, 2002; Heys et al., 2011; Ryan et al., 2012; Tierney et al., 2013), we consider a broader range of psychosocial factors which may be as or more important and are relevant to both men and women.

## Parenthood, Social Relations, and Cognitive Function

Social contacts facilitate the maintenance and development of cognitive functioning related to social interaction and the provision of social support which is hypothesized to buffer the harmful effects of stress (Seeman, Lusignolo, Albert, & Berkman, 2001). Consistent with this, evidence from longitudinal studies indicates that social contacts, strong social networks, and social participation are protective of cognitive function (Andel, Silverstein, & Kåreholt, 2014; Fratiglioni, Wang, Ericsson, Maytan, & Winblad, 2000; Seeman et al., 2011) and that sparse social interaction is associated with poorer cognitive functioning (Bassuk, Glass, & Berkman, 1999; Crooks, Lubben, Petitti, Little, & Chiu, 2008; Fratiglioni et al., 2000). Having children provides social stimulation both through interaction with them and because parenthood promotes involvement in other social relationships and activities (Knoester & Eggebeen, 2006). These associations between parenthood and social factors related to cognitive function may vary by gender. Some studies have found that childless men report higher levels of social isolation and depression than fathers, whereas childless women have more interaction with friends than mothers (Umberson, Crosnoe, & Reczek, 2010). In the present study, we therefore consider both women and men.

These factors mentioned above would suggest benefits for parents compared with nonparents, and possibly greater benefits for parents of more children. Our own previous research on older people in England for instance showed that parents had more regular face-to-face social contacts than their childless counterparts and that for mothers, number of children was positively associated with receipt of support (Grundy & Read, 2012). However, there may be some countervailing influences and certain types of parenting pathways which are less protective or indeed harmful. An early age at entry to parenthood and large family size (high parity) may lead to socioeconomic strain and limit opportunities for career involvement and progression, especially for women, which is relevant as occupational complexity is associated with later life cognition (Andel et al., 2014). Additionally “off-time” fertility may increase risks of depression and a poorer sense of control in life, partly due to associations with socioeconomic factors and possibly because of the reduced resilience of young parents to the stresses involved in raising children (Evenson & Simon, 2005; Koropeckyj-Cox, Pienta, & Brown, 2007; Read & Grundy, 2011). The timing of parenthood, and to some extent number of children, is socially patterned and strongly associated with levels of education. However, even taking account of this, previous studies suggest that early parenthood and high parity, as well as childlessness, are associated with poorer physical health in later life (Grundy & Read, 2015; Read, Grundy & Wolf, 2011). Lower socioeconomic position, poorer physical health, depression, and lack of control are all associated with poorer cognitive functioning (Agrigoroaei & Lachman, 2011; Brunner, 2005). Altogether, the prior evidence points to a need for more comprehensive consideration of socioeconomic, health, and social factors which may link fertility history to later life cognitive function. Here, we include a range of potentially negative and positive factors in our models in order to examine their role in the associations between fertility history and cognition in later life.

## Previous Research

Previous studies of associations between fertility history and cognitive function (reviewed below) have been motivated by the hypothesis that longer estrogen exposure is beneficial for cognitive function, which implies advantages for childless and low-parity women as estrogen is reduced during pregnancy. Two studies from China and the United States found that high parity was associated with lower (Heys et al., 2011) or greater decline in cognitive functioning in older women (McLay, Maki, & Lyketsos, 2003). Childlessness was conversely associated with better cognitive functioning (McLay et al., 2003). However, other studies have found no or different associations. These include studies of representative samples of French (Ryan, Carrière, Scali, Ritchie, & Ancelin, 2009) and Australian (Low, Anstey, Jorm, Rodgers, & Christensen, 2005) women and small or clinically based samples of women in Southern California (Smith et al., 1999), Canada (Hesson, 2012), and Korea (Kim, Stewart, Shin, & Yoon, 2003). In a Swedish study of older female twins, high parity was associated with cognitive impairment in a case–control analysis but not in a comparison of discordant twin pairs (Rasgon et al., 2005). In their French study, Ryan et al. (2009) also found that young age (under 21 years of age) at first birth was associated with poorer, and late motherhood (30+ years) with better cognitive performance. An association between older motherhood and better cognitive functioning in later life was also reported in a study of Chinese older women (Heys et al., 2011). However, a Korean study found no association between age at first birth and dementia (Kim et al., 2003). Conclusions from these investigations are thus inconsistent probably reflecting the range of study populations and designs used, the fact that several were based on clinical or otherwise nonpopulation representative samples, included only women and had limited control for relevant socioeconomic and psychosocial factors.

In this study, we investigate associations between parity and timing of the first and last birth with cognitive function among a population-based sample of adults aged 50+. We include women and men and a range of socioeconomic and social variables which may be associated both with fertility history and with cognitive function. Based on the sparse literature cited above and the broader literature on social interaction and associations between fertility history and health, we expected that both childlessness and high parity (four or more children) and, among parents, early age at entry to parenthood would be associated with poorer cognitive functioning later in life. As previous results on the effects of late parenthood are mixed, we had no explicit expectation on whether this would be positively or negatively associated with cognitive functioning. We expected that associations between parity, timing of births, and cognition might be partly explained by variations in socioeconomic position, health, and social participation.

## Method

### Sample

We used data from the English Longitudinal Study of Ageing (ELSA), a nationally representative longitudinal study of the older population of England. The first wave of ELSA, conducted in 2002–2003, included men and women then aged 50 years or older from private households which had participated in any one of the 1998, 1999, or 2001 rounds of the cross-sectional Health Survey for England (HSE), an annual government health survey of a stratified random sample of households. Response rates for the HSE were 69% in 1998, 70% in 1999, and 67% in 2001; the response rate for Wave 1 of ELSA was 67%. Comparisons with other sources, including census data, showed the baseline ELSA survey was nationally representative (Marmot, Banks, Blundell, Lessof, & Nazroo, 2003). Respondents have been reinterviewed every 2 years. We use data from the five first waves of ELSA for core members who provided an in-person interview in wave 1 (*n* = 11,233) to measure level and rate of change in cognition over an 8-year period. Tables 1 and 2 show the number of respondents with complete data for each variable used in the analysis.

**Table 1.** Distributions of Time-Invariant Variables at Wave 1 and Their Association With the Cognition Score Intercept (I), Linear Slope (S), and Quadratic Slope (Q) From the Latent Growth Curve Model Adjusted for Age and Gender

Variable	% or mean (*SD*)	*n*	Associations with cognition
Age in years (range 50–91^a^)	65.2 (10.35)	11,233	−0.04*** (I), −0.003*** (S), 0.001* (Q)
Educational qualifications		11,214	
Tertiary (diploma or degree)	22.1		Ref.
Secondary (O’ or A’ levels^b^)	21.8		−0.18*** (I)
Other qualification	13.5		−0.41*** (I)
No or lower qualification	42.6		−0.62*** (I)
Occupational status		10,770	
Professional/managerial	31.9		Ref.
Skilled nonmanual	24.1		−0.19*** (I)
Skilled manual	20.4		−0.42*** (I)
Unskilled or semiskilled manual	23.6		−0.53*** (I)
Home owner	80.0	11,181	0.31*** (I), 0.04* (S), −0.01* (Q)
Net wealth quintile (range 1–5)	3.0 (1.41)	11,134	0.13*** (I)
Smoking status		11,219	
Never smoked	35.6		Ref.
Current smoker	17.8		−0.15*** (I)
Past smoker	46.6		0.02 (I)
Physical activity	2.0 (0.88)	11,218	0.16*** (I)
Control (CASP-19) (range 1–4)	3.1 (0.67)	10,202	0.21*** (I)

*Notes*. ^a^Ages 91+ have been combined in one category to promote anonymity within the sample.

^b^Exams taken in high school at around age 16 (O’ level) and 18 (A’ level).

*p < .05, ***p < .001 (the estimates for the associations with I and S only shown when significant).

**Table 2.** Distributions of the Cognitive Score, Time-Varying Covariates, and Their Association With the Concurrent Cognitive Score (Cog.) in the Age- and Gender-Adjusted Latent Growth Curve Model

Variables	Wave 1			Wave 2			Wave 3			Wave 4			Wave 5		
	*n*	% or mean (*SD*)	Cog.	*n*	% or mean (*SD*)	Cog.	*n*	% or mean (*SD*)	Cog.	*n*	% or mean (*SD*)	Cog.	*n*	% or mean (*SD*)	Cog.
Cognitive score (range −2.9 to 2.8)^a^	11,040	0.00 (0.81)	—	8,620	−0.06 (0.85)	—	7,329	−0.09 (0.90)	—	6,366	−0.15 (0.95)	—	5,919	−0.22 (1.00)	—
Has limiting long-term illness	11,224	35.0	−0.08***	8,713	37.7	−0.06***	7,485	39.3	−0.07***	6,579	41.7	−0.09***	6,198	45.1	−0.09***
Depressive symptoms, CES- D8 > 2	11,040	24.7	−0.11***	8,588	25.0	−0.07***	7,304	24.6	−0.09***	6,344	24.5	−0.12***	5,890	27.2	−0.13***
Has partner	11,231	66.5	0.05***	8,718	64.6	0.08***	7,491	62.9	0.09***	6,582	61.1	0.10***	6,203	58.8	0.13***
Face-to-face contacts with child(ren) and/or family	10,145			7,469			6,236			5,447			5,280		
Once a week or more		59.4	Ref.		59.3	Ref.		59.2	Ref.		59.2	Ref.		58.6	Ref.
Monthly		29.9	0.08***		31.7	−0.02		32.3	0.06***		31.2	0.03*		32.0	−0.02
Less than once a month		10.7	0.06***		9.0	0.04***		8.5	0.04		9.6	0.02		9.4	0.01
Face-to-face contacts with friends	9,219			7,167			5,941			5,194			5,008		
Once a week or more		54.7	Ref.		56.2	Ref.		56.1	Ref.		56.8	Ref.		56.6	Ref.
Monthly		30.8	−0.02		36.8	−0.04**		37.0	−0.02		35.8	−0.03*		35.3	−0.01
Less than once a month		14.5	−0.07***		7.0	0.07**		6.9	−0.09**		7.5	−0.15***		8.1	−0.06**
Work/voluntary work	11,226	44.9	0.07***	8,736	45.4	0.02*	7,489	37.1	0.03**	6,581	31.8	0.03**	6,202	26.8	0.06***
Looking after home/family	11,226	42.7	0.03**	8,736	50.0	0.03***	7,489	42.4	0.02*	6,581	41.3	0.04***	6,202	40.0	0.07***
Caring for someone	11,226	9.6	0.04*	8,736	14.4	0.03	7,489	12.0	0.02	6,581	10.2	0.06**	6,202	10.3	0.02
Leisure activities (range 0–3)	9,201	1.5 (0.91)	0.18***	7,033	1.6 (0.86)	0.13***	5,668	1.6 (0.84)	0.11***	5,252	1.6 (0.85)	0.14***	5,083	1.6 (0.85)	0.24***
Social isolation (range 0–4)	10,168	1.0 (0.93)	−0.03***	7,713	1.1 (1.00)	−0.03***	6,405	1.1 (0.95)	−0.05***	5,541	1.1 (0.93)	−0.06***	5,437	1.2 (0.97)	−0.05***

*Note*. ^a^The mean is set at 0 in Wave 1. The coefficients for intercept = −0.00 (*SE* = 0.007), linear slope = −0.04 (*SE* = 0.006), quadratic slope = −0.004 (*SE* = 0.002), intercept variance = 0.31 (*SE* = 0.011), linear slope variance = 0.04 (*SE* = 0.008), quadratic slope variance = 0.003 (*SE* = 0.001). CES-D8 = Eight-item version of the Centre for Epidemiological Studies Depression Scale.

*p < .05. **p < .01. ***p < .001.

### Measures

We used three indicators of cognitive functioning selected because they were available in all waves and represent two important aspects of cognitive functioning—memory and executive functioning (Huppert, Gardener, & McWilliams, 2006). Memory was assessed using a word list recall test in which participants were asked to learn ten common unrelated words. Two scores were used: for immediate recall and for delayed recall which was assessed after letter cancellation and word fluency tasks. Executive functioning was measured using a verbal fluency test in which participants were asked to name as many different animals as possible in one minute. *Z*-scores for the three cognitive measures were calculated simultaneously for the five waves to allow mean levels to change over time. We used the mean of the *z*-scores as a combined cognitive index (average Cronbach’s alpha = 0.79, range 0.77–0.81). The distributions of both the original cognitive scores and the combined *z* scored were normal and continuous.

#### Fertility history

We derived fertility history variables from information collected in Wave 1. Number of children was measured using five binary variables indicating whether respondents had had 0, 1, 2, 3, or 4+ natural living children. For parents, we derived additional dichotomous variables indicating whether or not respondents had had a biological child before the age of 20 (women) or 23 (men) or after age 35 (women) or 39 (men). We chose these cut points on the basis of the previous literature and the distribution observed in the sample (Grundy & Tomassini, 2005).

### Covariates

We used time-invariant covariates from Wave 1 for variables which showed little or no change over time (Table 1). Age was measured in single years. Educational attainment identified whether respondents’ highest qualification was tertiary level (college or university diploma or degree); upper secondary (O’ or A’ levels or their equivalent—these are public examinations taken in secondary schools at around age 16 and 18, respectively); other (e.g., foreign or vocational qualifications); or whether they had no or lower level qualifications. Occupational status of respondents’ current or last job was classified into professional or managerial, skilled nonmanual, skilled manual, and unskilled or semiskilled manual groupings using the British occupational-based social class scheme (Galobardes, Shaw, Lawlor, Lynch, & Smith, 2006). Wealth and home-ownership were included as additional indicators of accumulated socioeconomic resources; these provide a better indicator of economic status in older age than current income (Banks, Breeze, Lessof, & Nazroo, 2008). Respondents were asked to report all financial wealth and estimate the value of other assets including housing, cars, and valuables such as jewelry and antiques. The summed value of these, net of debts, was divided into quintiles and treated as a continuous variable in the analysis. Tenure status indicated whether or not the respondent owned their home.

Smoking status was measured with three binary variables distinguishing current smokers, ex-smokers, and never-smokers. Self-reported physical activity included four categories: sedentary (no physical activity and, if working, in a sedentary job), low (mild physical activity at least once a week or if working in a job that was mostly standing), moderate (moderate physical activity at least once a week or if working in a job that involved physical work), and high (vigorous physical activity at least once a week or if working in a job that involved heavy manual labor) (de Oliveira, Shankar, Kumari, Nunn, & Steptoe, 2010). The distribution of physical activity was approximately normal and the association with outcomes was linear, so we treated this as a continuous variable. Control was measured with four items extracted from the CASP-19 quality of life questionnaire (Hyde, Wiggins, Higgs, & Blane, 2003). These items were: “My age prevents me from doing the things I would like to do”, “I feel that what happens to me is out of my control,” “I feel free to plan for the future,” and “I feel left out of things.” These were coded using a 4-point scale ranging from “often” to “never.” We reverse coded items so that a higher score indicates a higher sense of control and used the mean of the four items. The internal consistency of the scale was good (Cronbach’s alpha = 0.86).

Variables relating to health, social contacts, and activities were treated as time-varying; the distributions of these are shown in Table 2. Health variables comprised a binary indicator of self-reported long-term illness that limited activities (yes/no) and a short version of the Centre for Epidemiological Studies Depression Scale (CES-D) (Radloff, 1977). This scale included eight binary items so the count of depressive symptoms ranged from 0 to 8. Because the distribution was very skewed, depressive symptoms was dichotomized (0 = 0–2 symptoms, 1 = 3+ symptoms). We used a binary indicator of whether respondents currently had a partner (0 = no, 1 = yes). Face-to-face contacts with children/relatives and friends were measured using three binary items indicating contact less than monthly, between monthly and weekly, or weekly or more often. These variables were not linearly associated with cognitive functioning (see Table 2), fertility history, or other activity items (data not shown) so were included as separately rather than being summed into a score. We used three binary measures (coded 0 = no, 1 = yes) on activities in the past month: paid or voluntary work (including self-employment and work-related training); cared for a sick or disabled adult; and looked after home or family (homemaker). We also derived a count of three leisure activities: respondent currently had a hobby or pastime; was a member of any organization, religious group, or committee; and engaged in a cultural activity at least once a month (went to cinema, art gallery or museum, theatre, concert or opera). The social isolation score range from 0 to 4, with higher scores indicating greater social isolation. Positive scores were given if (1) respondents lived alone; (2) had less than monthly contact (of any kind) with child(ren) and/or other relatives or (3) friends; and (4) were not a member of any organization, religious group, or committee (de Oliveira et al., 2010). Although this score was somewhat skewed, its association with cognitive functioning was linear. We treated it as continuous because, as described below, maximum likelihood estimation can handle some nonnormality.

### Analysis

We used latent growth curve modeling to examine the level and rate of change in cognitive functioning and associations with fertility history. In this modeling, random effects are used to capture individual differences and fixed effects to estimate the average growth of the entire sample. Analyses were carried out using Mplus 7.11 (L. K. Muthén & M. B. O. Muthén, 1998–2012). Measurements collected at five time points were used to estimate the initial level (intercept) and linear and quadratic change in cognitive functioning. Models for number of children were fitted for all men and women. Models for early and late parenthood were fitted for mothers and fathers and adjusted for number of children. We added covariates in conceptually related steps. Thus, Model 1 included age; socioeconomic indicators were added in Model 2 (educational qualification, occupational social class, wealth, home-ownership) and physical and psychosocial health and health-related behaviors in Model 3 (long-term illness, smoking, physical activity, depressive symptoms, sense of control). The social engagement variables (contacts, activities, isolation) were partly overlapping and some associations were nonlinear and in different directions (see Table 2). For this reason, we first tested the positive dimensions of social engagement namely social contacts (Model 4a) and then additionally activities (Model 4b). We tested the negative dimension of social engagement (isolation) in Model 4c. Continuous covariates (age, wealth, physical activity, control, leisure activities, social isolation) were centered to make interpretation of the estimates easier. Model fit was assessed by chi-square analysis, but because this index is sensitive to sample size, we also used two other fit indices as recommended by Hu and Bentler (1999): the Comparative Fit Index (CFI) and Root Mean Square Error of Approximation (RMSEA) (Steiger, 1990). A value at or below 0.08 for the RMSEA and at or above 0.95 for the CFI is considered to indicate an acceptable model fit.

We used maximum likelihood estimation with robust standard errors (MLR) to take into account any nonnormality in the sample. Full information maximum likelihood was used. This method includes all respondents in the data regardless of whether they participated in the latter waves or responded to all items. The approach uses the information on mean and variance of the missing proportion of the variables given other observed variables. Using full information maximum likelihood is less biased and more efficient than listwise or pairwise deletion or similar response pattern imputation (Enders & Bandalos, 2001).

## Results

### Descriptive Results and Bivariate Associations Between Covariates and Cognitive Function

**Figure 1.**
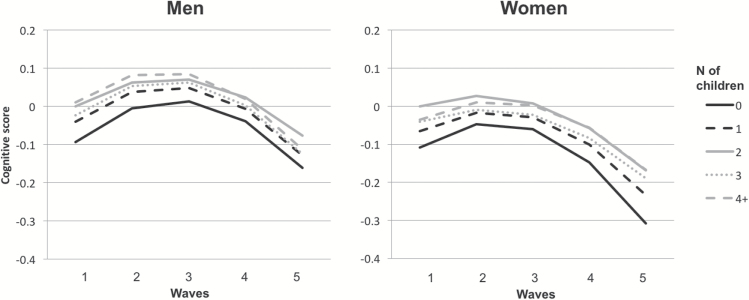
Cognitive functioning by parity (Model 4a, Table 3).

The sample included 6,123 women and 5,110 men. Of these, 16% were either childless or had one child, 38% had two children, 19% three children, and 12% four or more children. Thirteen percent of respondents had had an early age (<20 women/23 men) at entry to parenthood or a late age (>35 women/39 men) at last birth. Cognitive scores declined slightly over time (see Table 2 for the means and growth parameters). The rate of decline was initially slow and tended to become faster towards the end of the follow-up (Figure 1). Because cognitive functioning showed both linear and quadratic change over time, these growth terms were included in all subsequent models.

Table 1 shows the distribution of time-invariant covariates at Wave 1 and bivariate associations with cognitive functioning after adjustment for age and gender. The average age of respondents was 65. Twenty-two percent had either an upper secondary or tertiary level qualification and 80% were home owners. About one third were currently or previously employed in professional or managerial occupations, 44% in skilled jobs, and 24% in unskilled or semiskilled manual occupations. Fewer than 20% were current smokers and most people reported moderate physical activity and a sense of control at least sometimes. All these time-invariant covariates were associated with the initial level (intercept) of cognitive functioning: thus older age, lower socioeconomic position, current smoking, less frequent physical activity, and a lower sense of control were associated with lower levels of cognitive function. Age was also associated with the linear and quadratic slopes, indicating that the average rate of change (decline in cognitive functioning) was faster in older people. Home owners however retained their cognitive functioning longer at a higher level (slower rate of change). Associations were very similar for men and women and all in the same direction: 67 out of 77 (87%) estimate comparisons showed differences of 0.05 or less and similar strengths of evidence of association. The largest gender difference was found in associations between cognitive functioning and work and caring. In some waves, these associations were highly significant for women (*p* < .001) but borderline for men (*p* < .05–.10).

Table 2 shows the distribution of the time-varying covariates over the five survey waves and bivariate associations with cognitive function. About a third of respondents reported a limiting long-term illness and a fifth more than three depressive symptoms in Wave 1; these proportions increased over time. About two thirds had a partner initially and this proportion declined over time. More than half the sample had face-to-face contact with children or other relatives or friends at least once a week. Over time, there was a slight increase in proportions seeing relatives and friends less than once a week but at least monthly. Nearly half of the respondents initially undertook paid or voluntary work or were homemakers and about 10% reported caregiving. The proportion engaged in paid or voluntary work declined over time. Most people reported one or two out of three leisure activities and scored at least one on the social isolation scale. Most of the time-varying covariates showed the expected association with cognition: long-term illness, depressive symptoms, not having a partner, fewer activities, and social isolation were all associated with lower cognitive functioning. Associations between social contacts and cognition were more complex: a higher frequency of contacts with children and family was associated with poorer cognition (especially in the earlier waves), whereas a higher frequency of contacts with friends was associated with better cognition.

Those with no missing data were younger and had a higher socioeconomic position (results not shown). They reported average parity (two children), better health, more activities, and higher control. They had less frequent contacts with children and family and more frequent contacts with friends. They had a higher baseline level of cognitive functioning.

### Bivariate Associations Between Fertility History and Covariates

Associations between fertility history and the covariates (detailed results available from the authors) showed that high parity and early age at birth of the first child (<20 women/23 men) were associated with limiting long-term illness and depressive symptoms in women and with lower socioeconomic position, more adverse health-related behaviors, a lower sense of control, fewer activities, and more social isolation in both men and women. Childlessness was associated with lower socioeconomic position, more adverse health-related behaviors, depressive symptoms, and lower sense of control in men and fewer activities and more social isolation in both men and women. Early parenthood and childlessness were both associated with fewer contacts with relatives (including children where relevant). However, childlessness was associated with more frequent contacts with friends in men and women, whereas early motherhood was associated with less frequent contact with friends. Late age at last birth (>35 women/39 men) was to some extent associated with lower socioeconomic position in men and higher socioeconomic position in women and with slightly more adverse health-related behaviors and more frequent contacts with children and family in both genders.

### Fertility History and Level and Rate of Change in Cognitive Functioning

Table 3 shows the associations between fertility history and cognitive level. Models adjusted just for age indicated negative associations between high parity (four or more children for men and three or more children for women) and cognitive function (Model 1). However, these associations ceased to be significant when socioeconomic position was adjusted (Model 2). Having no child or one child compared to two children was also associated with a poorer level of cognitive functioning (Model 1). The association between having one child compared to two children and level of cognitive functioning substantially weakened (from −0.09 to −0.04 in men and −0.12 to −0.07 in women) when socioeconomic position was taken into account, showing no evidence of association in men after adjustment (Model 2) but remaining significant for women even after further adjustment (Models 2–4). In men, the effect of childlessness weakened after adjusting for socioeconomic status and health (from −0.16 to −0.08) but remained significant (Models 2–4). In women, associations between childlessness and an initial poorer level of cognitive functioning strengthened after adjusting for covariates, especially socioeconomic position (from −0.06 to −0.10) and social contacts (from −0.09 to −0.12) (Models 2 and 4a). Adding activities or using social isolation as an alternative negative indicator did not strengthen the association between childlessness and cognition or change any other estimates of associations between fertility history and cognition (Model 4b and 4c). In women, there was a persistent difference in the quadratic slopes such that childless women had a steeper decline in the last two waves of the study compared to women with two children (Figure 1).

**Table 3.** Associations Between Fertility History and the Initial Level of Cognitive Functioning in Men in ELSA Waves 1–5

	Models for men						Models for women					
	1	2	3	4a	4b	4c	1	2	3	4a	4b	4c
Number of children (ref. = 2)												
0^a^	−0.16***	−0.10***	−0.08**	−0.09***	−0.09***	−0.08**	−0.06*	−0.10***	−0.09***	−0.12***	−0.12***	−0.08**
1	−0.09**	−0.04	−0.04	−0.04	−0.04	−0.04	−0.12***	−0.07**	−0.06*	−0.07**	−0.06*	−0.06*
3	−0.05	−0.03	−0.03	−0.02	−0.02	−0.03	−0.09**	−0.04	−0.04	−0.04	−0.04	−0.05
4	−0.11***	0.00	0.01	0.01	0.01	0.01	−0.21***	−0.05	−0.04	−0.04	−0.03	−0.04
Early parenthood^b^	−0.06*	−0.01	−0.01	−0.01	−0.01	−0.02	−0.21***	−0.06*	−0.04	−0.04	−0.03	−0.05
Late parenthood^c^	−0.01	−0.01	0.01	0.01	0.01	−0.01	0.10***	0.07*	0.07*	0.07*	0.07*	0.06*

*Notes.* Model 1: adjusted for age; Model 2: +education, occupational status, tenure status, wealth; Model 3: +limiting long-term illness, physical activity, smoking, depressive symptoms, control; Model 4a: +partner, face-to-face contacts; Model 4b: + activities; Model 4c: Model 3 + social isolation. ELSA = English Longitudinal Study of Ageing.

^a^In all models for women, quadratic slope (−0.012*) was also significant.

^b^Parents only: early age at first birth (<20 women/23 men).

^c^Parents only: late age at last birth (>35 women/39 men).

*p < .05. **p < .01. ***p < .001.

Early age at entry to parenthood (<20 women/23 men) was associated with poorer cognitive level in women and men (Model 1), but after adjusting for socioeconomic position and health, the association disappeared (Models 2 and 3). Late parenthood (>35 women/39 men) was associated with a higher level of cognitive functioning in women. This association weakened after adjusting for socioeconomic position (from 0.10 to 0.07) but remained significant after adding the remaining covariates (Models 1–4c).

All models fitted the data well with CFI values >0.97 and RMSEA <0.02 (detailed results of model fit available from the authors). There were no differential effects (interactions) of age on the association between fertility history and the intercept, linear, or quadratic change in cognitive functioning.

## Discussion

In contemporary aging populations, age-associated cognitive decline presents one of the greatest challenges faced by individuals, families, and society (Deary et al., 2009). Improving understanding of life-course influences on cognitive function is therefore important. Previous studies have shown that socioeconomic factors, social interaction, and health are all associated with cognitive function (Agrigoroaei & Lachman, 2011; Brunner, 2005) and a separate body of research has identified links between these domains and fertility history and between fertility histories and later life health (Grundy & Read, 2015). However, the limited previous research into associations between fertility histories and later life cognitive function has paid little attention to the possible social pathways which may link them (Heys et al., 2011). The contribution of this paper is to partially fill this gap.

Our results show associations between number and timing of births and cognitive functioning in older age. Although a considerable amount of covariance was explained by socioeconomic factors, some of the associations remained even after adjusting for a number of factors related to health, depression, control, activities, social contacts, and isolation. Our results from models adjusted only for age showed a U-shaped association between parity and poorer cognitive functioning (Model 1 in Table 3) which is similar to associations between parity and physical health reported in previous studies (Grundy & Read, 2015; Grundy & Tomassini, 2005). In the case of high parity, the association with poorer functioning was largely due to a lower socioeconomic position among parents with large families and adding further controls for social interaction, depression, and lack of sense of control had trivial effects. This suggests that socioeconomic differences are an important driver of the association between high parity and poorer cognitive function and may partly operate through health and social engagement, although this requires further investigation.

In the case of associations between childlessness and cognitive functioning, the picture was rather different. For both men and women, childlessness remained associated with cognitive functioning even after adjustment for socioeconomic, health, and social engagement variables. Childless women also experienced faster cognitive decline towards the end of the follow-up. In the case of women, taking account of socioeconomic status and face-to-face contacts with children and/or other relatives strengthened, rather than weakened, the association between childlessness and poor cognitive functioning. This reflects the fact that a very high frequency of contacts with relatives was associated with poorer cognitive functioning (see Table 2), possibly because families initiate more contact with relatives who have cognitive problems. Moreover, childless women (contrary to childless men) were not socioeconomically disadvantaged.

Early parenthood was associated with poorer cognitive functioning but this association disappeared when socioeconomic position and health were taken into account. Other studies on early parenthood and various other health outcomes also point to a strong role of socioeconomic factors (Grundy & Read, 2015; Spence, 2008). We found no association between late fatherhood and cognitive functioning, to our knowledge, this has not been studied before. However, a higher age at last birth was associated with better cognitive functioning in mothers. This finding is consistent with some of the few previous studies which have examined this in women (Heys et al., 2011; Ryan et al., 2009) and with results on age at last motherhood and later physical health (Yi & Vaupel, 2004). Women having children later in life may benefit from social interactions with relatively young children or they may have had a higher cognitive level initially. Investigating this would require measures of cognitive functioning earlier in the life course.

### Limitations

We used high-quality nationally representative data to investigate associations between fertility histories and cognitive function, and change in cognitive function, in both women and men. The study nevertheless has some limitations. The numbers of older participants (70+) were relatively small and the study may have lacked sufficient power to identify differences by age group. Additionally, although we included a number of socioeconomic, health, and psychosocial covariates, some of which were time-varying, there may be some unobserved factors we could not control for which could usefully be investigated in future studies, for example, early-life influences and cognition in childhood or early adulthood. Attrition represents a further problem as older people who had initially lower cognitive scores were the most likely to be lost to follow-up. Moreover, these people were often childless or had a high number of children and had a lower socioeconomic position and poorer health and psychosocial functioning. Although the models took into account incomplete data and patterns of missingness, selection related to who initially participated may have reduced the variation in the sample and made it more difficult to detect associations between fertility history and cognition, especially change in cognitive functioning.

## Conclusions

Despite these limitations, this study has a number of strengths including use of a population-based sample of both women and men. This is a valuable addition, as most previous studies have focused on women and many have studied small samples, often not population based. As we show, there are numerous social pathways between fertility history and later life cognition affecting both men and women. Knowledge of these may be useful in planning and testing future interventions to promote maintenance of cognitive function.

There are also some methodological implications of our findings. Most previous studies have used a continuous measure for parity or a dichotomous measure of parenthood. Our study and those previous studies (Rasgon et al., 2005; Ryan et al., 2009) which have used categorical measures for parity have shown that associations are not linear. Moreover, our study highlights the importance of social factors. The association between parity and cognitive functioning turns out to be more complex when social contacts, activities, and isolation are taken into account, as there are nonlinear and differing directions of associations between the measures. This suggests that it is important to use the measures separately and allow for nonlinear associations.

To conclude, adverse effects of high parity and to some extent early childbearing and low parity appear to reflect underlying socioeconomic and health disparities. The poorer cognitive functioning of childless people suggests that there may be aspects of rearing children that are beneficial for cognitive function. For example, nurturance of others may promote self-esteem and self-efficacy and social interaction and activities with children, such as reading, playing games, and helping with homework, may be cognitively stimulating. Consistent with this hypothesis, one recent study of grandparent provision of care for grandchildren, which used an instrumental variable approach to allow for selection into this role, found an association between caring for grandchildren and grandparents’ verbal fluency (Arpino & Bordone, 2014). Given changes in fertility patterns, including increasing rates of childlessness, and other changes in patterns of social interaction, further work on linkages between family patterns, social interaction, and cognitive function is warranted.

## Funding

This work was supported by awards from the UK Economic and Social Research Council and National Institute for Health Research (grant number: ES/L001896/1) and a European Research Council Advanced Grant to Emily Grundy (reference number: 324055).
